# Convergent and discriminant validity of the Minimal Eating Observation Form – version II: a cross-sectional study

**DOI:** 10.1186/s12877-023-04639-x

**Published:** 2024-01-05

**Authors:** Albert Westergren, David Smithard, Mark Westergaard, Anne Norup, Johannes Riis, Anne Krarup, Line Elise Møller Hansen, Christina Emborg, Dorte Melgaard

**Affiliations:** 1 The PRO-CARE Group and the Research Platform for Collaboration for Health, Faculty of Health Sciences, Kristianstad University, SE-291 88, Kristianstad, Sweden; 2 Elderly Care, Queen Elizabeth Hospital, Lewisham and Greenwich NHS Trust, London, GB UK; 3 Centre for Exercise Activity and Rehabilitation (CEAR), School of Human Sciences, University of Greenwich, London, GB UK; 4 Department of Physiotherapy and Occupational Therapy, North Denmark Regional Hospital, Hjoerring, Denmark; 5 Department of Geriatric Medicine, Aalborg University Hospital, Aalborg, Denmark; 6 Department of Acute Medicine and Trauma Care, Aalborg University Hospital, Aalborg, Denmark; 7 Faculty of Clinical Medicine, Aalborg University, Aalborg, Denmark; 8 Mech-Sense, Department of Gastroenterology and Hepatology, Aalborg University Hospital, Aalborg, Denmark; 9 Nutritional Team, Roskilde Municipality, Denmark

**Keywords:** Convergent, Discriminant, Dysphagia, Eating difficulties, Validity

## Abstract

**Background:**

The Minimal Eating Observation Form – Version II (MEOF-II) is a brief and easy to use screening tool for eating difficulties, that is psychometrically robust. The aim of this study was to explore convergent (measuring similar constructs) and discriminant (measuring somewhat different constructs) validity of the MEOF-II to other validated dysphagia specific, activity and participation related instruments.

**Methods:**

In this cross-sectional study, participants (*n* = 100, mean age 72, *n* = 42 women), diagnosed with either chronic pulmonary disease, Parkinson´s disease, Multiple Sclerosis, or stroke were recruited from rehabilitation centres. Patient-reported outcomes and clinical-rated assessments, capturing eating ability in general and swallowing in specific, included: The Dysphagia Handicap Index (DHI)*,* the 4-question test (4QT), the Minimal Eating Observation Form – II, the Volume – Viscosity Swallow Test (V-VST), Flexible Endoscopic Evaluation of Swallowing (FEES) documented according to the Penetration-Aspiration Scale (PAS). Type of oral intake was documented using the Functional Oral Intake Scale (FOIS). Activities in daily living was assessed with Barthel index (BI). Spearman’s correlation coefficient was used to analyze associations. The MEOF-II total score was hypothesised to have moderate correlations (*r* ≥ 0.3) with the other assessments, besides with PAS and FOIS (weak correlations, *r* < 0.3).

**Results:**

In total 78 participants had any type of eating difficulties (MEOF-II), 69 reported dysphagia (4QT), 62 had dysphagia according to V-VST, 29 showed evidence of penetration/aspiration (PAS), and 31 participants had decreased oral intake ability (FOIS). The MEOF-II total score had moderate correlations with DHI, BI, 4QT, V-VST volume, and weak correlations with V-VST dysphagia and viscosity, PAS, and FOIS. Comparing a prior hypothesised correlation strengths against empirical findings showed that 83% of the hypothesised correlations were correct.

**Conclusions:**

The MEOF-II is a holistic and objective screening tool that can indicate the need for further assessment and corresponds well with the persons’ subjective experiences. MEOF-II does not specifically assess the risk for penetration/aspiration.

**Supplementary Information:**

The online version contains supplementary material available at 10.1186/s12877-023-04639-x.

## Background

When screening a patient´s eating ability we can identify eating difficulties needing further assessment or treatment whereby the patients´ quality of life can be increased. The Minimal Eating Observation Form – version II (MEOF-II) has been developed for this screening purpose. The MEOF-II can be completed even if the patient is severely disabled. It is easy and fast to complete and requires a minimum of training, and measure eating as an activity, the ability to eat. It does not focus specifically on social interactions and the value of the eating experience [1]. The MEOF-II has been evaluated in several studies [2–4], been described as high-quality evidence in reviews [5, 6], and been used in a number of other studies [7–11]. However, it´s convergent and discriminant validity has only been evaluated to a limited extent. Convergent validity focusses on weather different measures assess the same construct while discriminant validity focusses on weather different measures assess different constructs [12]. Thus, the focus here is on the MEOF-II validity as compared to other measures, some easy to use and other more specialized and even invasive.

Previous studies using the MEOF-II have shown the prevalence of eating difficulties to range from 55–82% in different settings [7, 9, 10]. Eating difficulties are associated with malnutrition [11], low Body Mass Index, weight loss, eating assistance, provision of energy-enriched food, supplements, and modified consistency of diet [2]. Further on, among patients with stroke, in geriatric patients as well as among patients with pneumonia, eating difficulties are associated with longer length of hospital stay, and the need for discharge to nursing homes [13–15]. Finally, eating difficulties increase the risk of having low activity of daily living function (ADL) or of having poor nutritional status within geriatric patients [7]. It is vital to diagnose eating difficulties in order to start the needed treatment as soon as possible to increase the patient’s quality of life and to avoid aspiration pneumonia [16]. However, testing for eating difficulties is often time consuming, expensive or requires that the patient is tested at a hospital. Several instruments measuring different aspects of eating difficulties exist, but few of them are as feasible to use in everyday care or even in a patients’ own home as the MEOF-II. No studies have yet focused on whether the MEOF-II might be able to measure the same aspects of dysphagia as the instruments used in the clinic. If it does, then more patients can be tested even in their own home, and some could be spared hospital visits and extensive testing of objective aspects of dysphagia. We hypothesised that the MEOF-II screening tool possibly could “stand alone” in clinical assessment of dysphagia. The aim of this study was to explore convergent (measuring similar constructs) and discriminant (measuring somewhat different constructs) validity of the MEOF-II to other validated dysphagia specific and ADL related instruments, in rehabilitation patients, and in four medical conditions.

## Methods

This cross-sectional study was conducted according to the declaration of Helsinki, and carried out in the North Denmark Regional Hospital, Hjørring, Denmark. The North Denmark Regional Ethics Committee on Health Research Ethics approved the study (approval number: N-20210026). All the participants gave oral and written informed consent before study start. This study is reported according to the COSMIN reporting guideline [17].

### Participants

This study recruited participants in the community via newspaper advertisement and rehabilitation centres in different municipalities. Participants were included if they were diagnosed with chronic obstructive pulmonary disease (COPD), Parkinson’s disease (PD), Multiple Sclerosis (MS) or had sequelae after stroke. These diagnostic groups were chosen since eating difficulties in general and dysphagia in specific are common among these groups [18–21]. The participants should have an age of ≥ 18 years, speak and understand Danish, and have a sufficient energy level to perform the tests. The exclusion criteria were cognitively impaired (could not give informed consent) or had severe dysphagia (i.e. using a feeding tube). Three participants were excluded: two due to severe cognitive impairment and one due to not fulfilling the inclusion criteria according to diagnosis. Participants were included until we reached about 25 participants in each of the four diagnosis groups.

### Data collection

Demographic data (age, sex, height, weight, BMI, comorbidities) were collected by interview. All the participants completed standardized questionnaires – *The Dysphagia Handicap Index* (DHI)*, Barthel index* (BI) and *4 Questionnaire Test* (4QT). Each individual test was carried out by one and the same trained assessor on all participants. For the different tests, there were different assessors. The participants completed three previously validated clinical tests – *The Minimal Eating Observation Form – II* (MEOF-II), *Volume – Viscosity Swallow Test* (V-VST), *Flexible Endoscopic Evaluation of Swallowing* (FEES) documented according to the *Penetration-Aspiration Scale* (PAS). The FEES was conducted by a dysphagia therapist, who is experienced in performing FEES in intensive care unit patients and geriatric patients, he also did the PAS scoring. Type of oral intake was documented using the *Functional Oral Intake Scale* (FOIS). Four different investigators performed the questionnaires and tests – the first was responsible for the questionnaires, the second for MEOF-II, the third for V-VST, and the last for the FEES-test. All the clinical tests were performed by trained occupational therapists experienced in assessment of dysphagia, and in a randomized order, and the investigators were blinded. The assessment of MEOF-II and V-VST was conducted separately by two occupational therapists specialized in dysphagia and by a single examiner for each test to ensure consistency.

The questionnaires and the clinical tests cover aspects of body *function* (swallowing function), *activity* (the ability to eat), as well as of *participation* (to eat together with other persons) [22]. Some of the tests have a broad focus and include activity and body function, such as the MEOF-II and even participation as in the case with the DHI. Other tests have a narrow focus on body function, such as the V-VST and the FEES.

### Questionnaires

*DHI* is a validated patient-reported questionnaire, which can be used to evaluate the effect and severity of dysphagia from the patient’s perspective [23, 24], and covers broad aspects including body function, activity, and participation. In 25 items, the questionnaire reflects the emotional (7 items, score range 0–28), physical (9 items, score range 0–36), and functional (9 items, score range 0–36) aspects of dysphagia. There are three response options for each question: never, sometimes, or always. In the same questionnaire, the participants are asked to rate, their swallowing problems on a scale from one to seven. There are verbal descriptors for three of the seven response options: for one “no difficulty at all”; for four “somewhat of a problem”; and for seven “the worst problem you could have”. In addition, there is one single item, where the respondent is asked to circle the severity of his/her swallowing difficulty from one to seven. The three verbal descriptors are “normal” (one), “moderate problems” (four) and “severe problems” (seven) [23, 24]. The total score, subdomain scores, and single item score were used in the analysis.

*BI* is an instrument used to evaluate functional ability based on 10 different functional areas such as personal hygiene and feeding, and thus mainly captures activities. The instrument is useful to evaluate the patients need for assistance in their daily living. The ten items have two to four response options and generate a score range between 0–100, where higher score indicates less problems with activities of daily living [25, 26]. The total score was used in the analysis.

*4QT* is a four-item questionnaire for screening the older adults for dysphagia [27], and mainly captures the body function swallowing, but also to some extent the activity to eat. The items are:

- 1) Do you cough and choke when you eat and drink?
- 2) Does it take longer to eat your meals than it used to?
- 3) Have you changed the type of food that you eat?
- 4) Does your voice change after eating/drinking?

Participants were asked to answer the four items with the response categories: zero = no problem; and one = having problem. The instrument generates a score range from zero to four, where higher score indicates more problem. The total score was used in the analysis.

### Clinical tests

*MEOF-II* is a screening tool for the structured observation of eating difficulties within the categories: ingestion; deglutition; and energy/appetite. Thus, it captures mainly eating as an activity as well as swallowing as a body function. Each category contains three sub-questions and a rate of zero indicates normal eating, whereas a rate of one indicates an eating difficulty. MEOF-II allows for score summaries within each category as well a total-score, including all nine items [4]. Thus, the category scores range from 0 to 3, and the total score from 0 to 9, where lower scores indicate no/less problems. MEOF-II is psychometrically robust, including for instance good validity and inter-rater reliability [2–4, 28, 29]. Reliability, i.e. internal consistency, has been assessed using the MEOF-II, resulting in a Cronbach's alpha of 0.79 among trained observers, and 0.71 among less trained observers [2]. High exact agreement between raters (inter-rater reliability) was noted at 0.89 (kappa 0.70) [2]. In a Chinese context, the inter-rater reliability, measured by Pearson's rho, was 0.79, while intra-rater reliability (same rater at different occasions) was established at 0.96 [29]. The total score and the category scores were used in the analysis.

*V-VST* is a bedside swallowing test, which is used to evaluate the safety and efficacy of swallowing different liquids (nectar, water and pudding) in three different volumes (5 ml, 10 ml and 20 ml). V-VST has a narrow focus on the body function “swallowing”. Thickener Resource ThickenUp (Nestle´ HealthCare Nutrition) was used to obtain the nectar and pudding consistence. Changes in the voice, a drop in oxygen saturation ≥ 3% and cough indicate a decreased safety in swallowing and could indicate an increased risk of aspiration [30, 31]. Thus, impaired safety, i.e. coughing and a decrease in oxygen saturation was recorded as having dysphagia (no = 0; yes = 1). What viscosity that was used was also recorded (unmodified = 0; nectar = 1; pudding = 2), as well as what volumes that the patient managed to swallow, safe and efficient (high [20 ml] = 0; middle [10 ml] = 1; and low [5 ml] = 2). Thus, higher scores in all three aspects indicates worse problems, and the actual score for each item, as described above, were used in the analysis.

*FEES.* A laryngoscope was passed through the nose to evaluate the pharyngeal and laryngeal anatomical structure and the swallowing function [32, 33]. Thus, it has a narrow focus on the body function. An Olympus ENF-P3 laryngoscope attached to a CCD camera and a colour monitor was used. The participants were given three different liquids (nectar, water and pudding) in three different volumes (5 ml, 10 ml and 20 ml). The same Thickener Resource ThickenUp (Nestle´ HealthCare Nutrition) as used in V-VST was used to obtain nectar and pudding consistence. To detect the liquid more clearly, the liquid was colored with a blue food dye. Any cough, or sign of penetration or aspiration was noted. The results were documented according to *PAS* on a scale ranging from one to eight, with verbal descriptions for each response category (1 = material does not enter the airway, 8 = material enters the airway, passes below the vocal folds, no effort made to eject) [34, 35]. Thus, higher scores indicate worse problems, and the actual PAS score was used in the analysis.

*FOIS* reflects the functional oral intake of patients with dysphagia on a scale from 1–7, with verbal descriptors for each response category (1 = nothing by mouth, 7 = total oral intake without restrictions [36, 37]. Thus, lower scores indicate worse problems. FOIS has a narrow focus relating to the body function “swallowing”. The actual FOIS score was used in the analysis.

### Analyses

Data were checked regarding underlying assumptions and described and analyzed accordingly using IBM SPSS 24 (IBM Corp., Armonk, NY). The alpha level of significance was set at 0.05 (2-tailed). Spearman’s correlation coefficient was used to analyze associations between MEOF-II, total score, and subconstruct scores. With a sample of 100 we considered spearman correlations of *r* < 0.3 as weak, *r* 0.3–0.6 as moderate, and *r* > 0.6 as strong [38]. To detect a correlation coefficient of at least 0.3, at a two tailed alpha level of 0.05, with sufficient power (80%) the minimum required sample size is *n* = 84 [39].

### Hypotheses for the validity analysis

Convergent validity is generally considered acceptable if more than 75% of hypothesis are correct, and correlations with related (convergent validity) constructs should be higher than with unrelated (discriminant validity) [12]. Since some instruments had a broad focus (eating as an activity, swallowing as a body function, and participation when eating) and some had a narrow focus (swallowing as a body function), no high correlations (*r* > 0.6) were specifically expected. In addition, it is well known that clinician-rated and patient-reported outcomes, although measuring the same construct, may lead to somewhat different results. For instance, when it comes to dysphagia, more patients reported having signs/symptoms of swallowing dysfunction than what was found in objective swallowing tests among patients with COPD [19], and among patients with MS [20]. While among patients with PD the relationship was the opposite, thus the prevalence was higher with objective testing compared to the subjective reports [21]. Thus, one cannot expect high correlations between all the tools used in this study. Thus, it was hypothesized that there would be weak associations between the MEOF-II category intake and the other assessments, except with BI. The MEOF-II category swallowing was expected to have moderate correlations with the other assessments. The MEOF-II category energy/appetite was expected to have a mix of moderate (4QT, DHI and its dimensions) and weak associations (V-VST, BI, PAS and FOIS). The MEOF-II total score was expected to have moderate associations with the other assessments, besides with PAS and FOIS (Table 1).

**Table 1:** Hypothesized associations between MEOF-II and other factors for a sample size of *n* = 100

	Direction of correlation	**MEOF-II**			
		**Intake**	**Swallowing**	**Energy/appetite**	**Total score**
Dysphagia Handicap Index (DHI)					
Physical	+	Weak	Moderate	Moderate	Moderate
Functional	+	Weak	Moderate	Moderate	Moderate
Emotional	+	Weak	Moderate	Moderate	Moderate
Total score	+	Weak	Moderate	Moderate	Moderate
Dysphagia Handicap self-rated	+	Weak	Moderate	Moderate	Moderate
Activities of daily living (Barthel index)	-	Moderate	Moderate	Weak	Moderate
Dysphagia (4QT)	+	Weak	Moderate	Moderate	Moderate
Volume viscosity swallow test (V-VST)					
Dysphagia	+	Weak	Moderate	Weak	Moderate
Viscosity	+	Weak	Moderate	Weak	Moderate
Volume	+	Weak	Moderate	Weak	Moderate
Penetration-Aspiration Scale (PAS)	+	Weak	Moderate	Weak	Weak
Functional Oral Intake Scale (FOIS)	-	Weak	Moderate	Weak	Weak

Hypothetical interpretation of spearman correlations: Weak: *r* < 0.3; Moderate/strong: *r* ≥ 0.3

## Results

### Characteristics of the sample

In Table 2 the characteristics of the total sample (*n* = 100), as well as characteristics of the subsamples (stroke (*n* = 25), COPD (*n* = 25), MS (*n* = 24), and PD (*n* = 26)) are described. In total 62 patients (62%) had dysphagia according to the VVST and it was least common among patients with MS (40%). Penetration/aspiration (PAS) was found in 29% and it was least common amongst patients with MS (20%). Patients with PD reported worse total score in DHI compared to the other subsamples. In the total sample 78% had difficulty eating, according to MEOF-II, and in the MS subsample 62%, while it was 81 to 84% in the other three subsamples (Table 2).

**Table 2:** Patient characteristics, all patients, patients with stroke, COPD, MS and PD respectively

	**All patients, *n* = 100**	**Stroke, *n* = 25**	**COPD, *n* = 25**	**MS, *n* = 24**	**PD, *n* = 26**
**Age**, median (q1-q3)	72 (63–77)	68 (58–75)	75 (70–78	68 (53–74	75 (69–78)
**Female/male**, n	42/58	6/19	15/10	15/5	6/20
**Body Mass Index**, median (q1-q3)	26.4 (22.8–30.5)	26.0 (21.9–28.1)	26.3 (21.8–31.0)	27.9 (23.3–30.7)	25.7 (23.5–31.0)
**Activities of daily living,** high score = better, median (q1-q3)	90 (60–100)	85 (42–100)	95 (87–100)	90 (62–95)	90 (70–100)
**Dysphagia Handicap Index,** low score = better, median (q1-q3)					
Physical	8 (4–14)	8 (4–12)	10 (4–18)	8 (4–11)	10 (6–15)
Functional	6 (2–12)	4 (0–13)	8 (2–20)	4 (0–8)	12 (4–14)
Emotional	4 (0–8)	4 (0–8)	4 (0–12)	4 (0–9)	4 (0–6)
Total score	20 (8–34)	20 (4–32)	18 (8–47)	16 (6–28)	28 (12–40)
**Dysphagia Handicap self-rated,** low score = better, median (q1-q3)	3 (1–4)	2 (1–4)	3 (1–4)	2 (1–4)	3 (2–4)
**MEOF-II, low score = better)**					
Food intake, median (q1-q3)	0 (0–1)	1 (0–2)	0 (0–0)	0 (0–1)	0 (0–1)
Food intake problems, n	32	12	4	7	9
Swallowing, median (q1-q3)	0 (0–2)	0 (0–2)	0 (0–2)	0 (0–1)	0 (0–1)
Swallowing problems, n	38	11	9	9	9
Energy/appetite, median (q1-q3)	1 (0–1)	1 (0–2)	1 (1–1)	1 (0–1)	1 (0–1)
Energy/appetite problems, n	64	13	20	13	18
Total score, median (q1-q3)	2 (1–4)	2 (2–4)	1 (1–4)	1 (1–2)	2 (1–4)
Any problems, n	78	21	21	15	21
**Dysphagia,** 4QT, low score = better, median (q1-q3)	2 (0–3)	1 (1–1)	1 (0–1)	1 (0–1)	1 (1–1)
Any problems, n	69	17	19	12	21
**Volume-viscosity swallow test** (V-VST)					
Dysphagia, n	62	17	17	10	18
Viscosity, unmodified/nectar/pudding, n	89/7/1	22/1/1	21/4/0	23/1/0	23/1/0
Volume, high (20 ml)/middle (10 ml)/low (5 ml), n	63/26/8	16/8/0	12/8/3	19/4/1	14/6/4
**Penetration-Aspiration, PAS, low score = better,** median (q1-q3)	1 (1–1.2)	0 (0–1)	0 (0–1)	0 (0–0)	0 (0–0)
Having penetration/aspiration, n	29	10	8	5	6
**Oral Intake,** FOIS, high score = better, median (q1-q3)	7 (6–7)	0 (0–1)	0 (0–1)	0 (0–0)	0 (0–0)
Decreased oral intake ability, n	31	10	10	5	6

*COPD* Chronic Obstructive Pulmonary Disease, *MS* Multiple Sclerosis, *PD* Parkinsons Disease

### MEOF-II validity

**Fig. 1:**
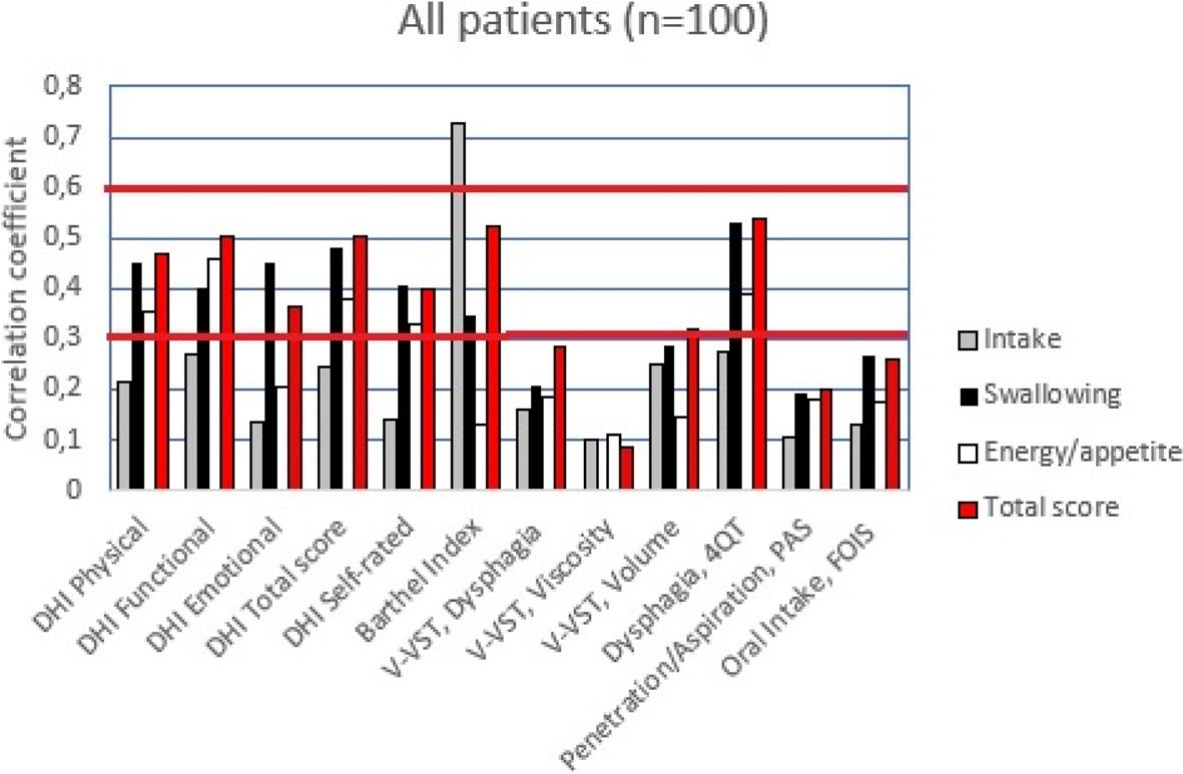
Spearman correlations coefficients between the MEOF-II (Minimal Eating Observation Form – Version II) categories: intake; swallowing; energy/appetite; and total score. Illustrated for the total sample. Red thicker horizontal lines marks Spearman correlations: weak = *r* < 0.3; moderate = *r* 0.3–0.6; strong = *r* > 0.6

Comparing our hypothesis against empirical findings showed that eight (17%) of the hypothesis were wrong and 40 (83%) were correct. Unexpectedly (wrong hypothesis) we found only weak associations between V-VST (dysphagia, viscosity), PAS and FOIS in relation the MEOF-II category swallowing. In addition, also unexpectedly, there were only weak associations between V-VST (dysphagia, viscosity, volume) in relation to the MEOF-II total score. Also, there was only a weak association between DHI (emotional score) and the MEOF-II category energy/appetite (Fig. 1 and Table 3).

**Table 3:** Correlations between Minimal Eating Observation Form – version II (MEOF-II) and different clinical characteristics (*n* = 100)

	**MEOF-II**							
	**Intake**		**Swallowing**		**Energy/appetite**		**Total score**	
**Dysphagia Handicap Index (DHI)**	***r***	^a)^	***r***	^a)^	***r***	^a)^	***r***	^a)^
Physical	.215	Weak	.451	Moderate	.353	Moderate	.471	Moderate
Functional	.270	Weak	.400	Moderate	.458	Moderate	.501	Moderate
Emotional	.133	Weak	.452	Moderate	**.207**	**Weak**	.366	Moderate
Total score	.245	Weak	.479	Moderate	.380	Moderate	.505	Moderate
**DHI self-rated**	.140	Weak	.404	Moderate	.329	Moderate	.401	Moderate
**Activities of daily living (Barthel index)**	-.727	Strong	-.345	Moderate	-.129	Weak	-.522	Moderate
**Dysphagia (4QT)**	.274	Weak	.531	Moderate	.387	Moderate	.536	Moderate
**Volume-viscosity swallow test (V-VST)**								
Dysphagia	.159	Weak	**.208**	**Weak**	.183	Weak	**.286**	**Weak**
Viscosity	.102	Weak	**.003**	**Weak**	.111	Weak	**.085**	**Weak**
Volume	.251	Weak	**.287**	**Weak**	.147	Weak	.318	Moderate
**Penetration-Aspiration Scale (PAS)**	.105	Weak	**.190**	**Weak**	.182	Weak	.201	Weak
**Functional Oral Intake Scale (FOIS)**	-.132	Weak	**-.269**	**Weak**	-.175	Weak	-.260	Weak

Bold correlation coefficients indicate deviation from the hypothesis

^a)^Spearman correlations: weak = *r* < 0.3; moderate = *r* 0.3–0.6; strong = *r* > 0.6

The associations between eating difficulties (according to MEOF-II) BI and the eating related measures in the different subsamples are illustrated in Supplementary file, and the exact correlation coefficients are also given in Supplementary file.

### Stroke

The associations between MEOF-II total score and the other indices among patients with stroke, showed a strong association (*r* > 0.6) with BI, moderate (*r* = 0.3–0.6) with DHI (physical, functional, and total score) and the 4QT. Otherwise the associations were weak (Supplementary file).

### COPD

The associations between MEOF-II total score and the other indices among patients with COPD, showed strong associations (*r* > 0.6) with DHI functional score and the 4QT, moderate (*r* = 0.3–0.6) with DHI (physical, emotional, total score, and self-rated), BI, V-VST (dysphagia, and volume). Otherwise, the associations were weak (Supplementary file).

### MS

The associations between MEOF-II total score and the other indices among patients with MS, showed strong associations (*r* > 0.6) with DHI (total score, and self-rated), and the 4QT, moderate (*r* = 0.3–0.6) with DHI (physical, functional, and emotional), BI, V-VST (dysphagia, and volume), PAS, and FOIS. Otherwise, the associations were weak (Supplementary file).

### PD

Finally, among patients with PD, the associations between MEOF-II total score and the other indices, showed no strong associations (*r* > 0.6), moderate associations (*r* = 0.3–0.6) with DHI (physical, functional, emotional, total score, and self-rated), BI, V-VST (volume), and 4QT. Otherwise the associations were weak (Supplementary file).

## Discussion

The MEOF-II measures the similar areas as the questionnaires DHI, 4QT, and BI (high convergent validity). It does not measure the same as the clinical tests V-VST, PAS, or FOIS. As expected, correlations between the MEOF-II intake, or energy/appetite components did not correlate well with V-VST, PAS, and FOIS (discriminant validity). Surprisingly, the correlations were weak between MEOF-II swallowing component and V-VST, PAS, and FOIS.

By comparing our hypothesis with the empirical findings, we measured a new validity aspect of the MEOF-II – convergent and discriminant validity. As a rule of thumb, convergent validity is generally considered adequate if more than 75% of the hypotheses are correct [12], and here it was 83%. However, somewhat surprising the share of correct hypothesis was only 42% between the MEOF-II swallowing component and the other assessments (V-VST, PAS, FOIS). When a hypothesis is wrong, this can have several causes: (1) the instrument under examination does not measure what it is supposed to, (2) the comparator instrument does not measure what it is supposed to, or (3) the theory or assumptions underlying the hypothesis are incorrect [40]. In this study, we believe it was the assumptions underlying the hypotheses that were incorrect. Thus, the unexpected finding might be because the MEOF-II swallowing component measures broader aspects of “swallowing” e.g. the ability to manipulate the food in the mouth, swallow, and chew where to the comparators V-VST and PAS measures how food consistency affects swallowing. These discrepancies between a broad versus a narrow focus can contribute to the weak correlations. Thus, in retrospect, the low correlations might not be that surprising. The findings also indicate that the MEOF-II screening needs to be combined with some other assessment of the risk for penetration/aspiration.

There are four main reasons for why MEOF-II can be recommended to be used in clinical practice.

First, MEOF-II has a broad holistic perspective on eating, in line, as indicated by moderate correlations, with the perspectives of DHI and 4QT. These findings indicates that problems objectively identified by MEOF-II also matters for the patients’ subjective experiences. This is important, since MEOF-II focus on measuring eating as an activity and not on social interactions (participation) and the value of the eating experience per see [1]. Further on, the MEOF-II intake scores correlated strongly with BI, as expected since “feeding” is one of the items in the BI [25]. Second, it seems valid to use the MEOF-II when screening for eating difficulties since it is based on observation and can be used even if patients have difficulties communicating and/or are cognitively impaired. Third, if only using very specific assessments like PAS, FOIS, or FEES that focus entirely on dysphagia, other problems of relevance for the eating ability might be missed, and not taken care for. Fourth, making observations based on the MEOF-II requires minimal training [2]. But, if only using the MEOF-II, aspiration/penetration might be missed, since there was only a weak association between MEOF-II swallowing component and the PAS. Thus, the MEOF-II seems to be a good overall screening tool for the need of treatment, and in cases where aspiration or penetration is suspected, the MEOF-II needs to be followed by a FEES or V-VST which measures that aspect better. The tricky part is how to identify patients in need for FEES. However, this was not within the scope of this study, but will be relevant to uncover in future studies.

Although the objective was not to explore the impact of age and gender it is worthwhile to briefly discuss. First, the MEOF-II has been found to work invariantly (no Differential Item Functioning) between men and women, and between younger and older [4]. Second, having eating difficulties is associated with needing eating assistance. For instance, among general geriatric patients 62% had eating difficulties but could eat without assistance, and significantly more, 97% had eating difficulties among those needing assistance [18]. Eating assistance was found to be significantly more common among women (40%) than among men (34%), and among older (70 years and over, 42%) than among younger (below 70 years, 14%) [2]. Thus, gender and age might have some impact on the achieved MEOF-II scores, although the tool itself, from a measurement perspective, works invariantly across gender and age.

Although patients with cognitive impairments were excluded from this study, it should be noted that the MEOF-II can be used also for this group of patients. Since all items but one is based on observations during eating it works also for cognitively impaired patients. The question that is not based on observation is the question about appetite now compared to before. In the MEOF-II manual the question about appetite should on the first hand be answered by the person him-/herself and if not possible the assessor needs to do a reasonable estimation of the appetite based on once knowledge about the patient.

There are strengths and limitations with this study. It is a strength that the sample size of the total sample had enough power to detect correlations above 0.3. Further we included patients having different conditions increasing the generalisability of our results across patient groups. However, it is a limitation that the sub-samples were rather small [39]. The overall design was strong, using blinding with different assessors and a heterogenous patient group to increase generalisability. It is also a strength that patient reported outcomes were tested in combination with objective observations which are less invasive, as well as with FEES. Observations that are more objective than a questionnaire alone. Another strength is that MEOF-II was measured up against many previously validated tests. It is also a strength that the hypotheses are clearly expressed and tested, to increase transparency.

## Conclusion

The findings provide additional support for the validity of the MEOF-II as compared to related instruments. The MEOF-II is a holistic overall screening tool for observing the ability to eat a meal, that can indicate the need for further assessment. MEOF-II measures aspects of eating that are of importance for the patients. It covers similar aspects as the DHI, BI and 4QT. However, MEOF-II does not measure penetration/aspiration well and should be combined with better instruments for this purpose e.g. V-VST and/or FEES.

### Supplementary Information

**Additional file 1.**

## Data Availability

The dataset analysed during the current study is available from the corresponding author on reasonable request.

## Abbreviations

BI: Barthel Index
DHI: Dysphagia Handicap Index
FEES: Flexible Endoscopic Evaluation of Swallowing
FOIS: Functional Oral Intake Scale
MEOF-II: Minimal Eating Observation Form – Version II
MS: Multiple Sclerosis
PAS: Penetration-Aspiration Scale
PD: Parkinson´s disease
V-VST: Volume-Viscosity Swallow Test
4QT: 4-Question test
