# Supplementary material for: Convergent and discriminant validity of the Minimal Eating Observation Form – version II: a cross-sectional study
Source: BMC Geriatr. 2024 Jan 5;24:27. doi: 10.1186/s12877-023-04639-x (PMC10770885; doi:10.1186/s12877-023-04639-x)
Supplement: Supplementary file 1 — Additional file 1. [file 12877_2023_4639_MOESM1_ESM.docx]

Figure 1. Spearman correlations coefficients between the MEOF-II (Minimal Eating Observation Form – Version II) categories: intake; swallowing; energy/appetite; and total score. Illustrated for patients with stroke, COPD = Chronic Obstructive Pulmonary Disease; MS = Multiple Sclerosis; and PD = Parkinsons Disease.

Table 1. Correlations between Minimal Eating Observation Form – version II (MEOF-II) and different clinical characteristics among patients with stroke (n=25).

|  | **MEOF-II** | | | | | | | |
| --- | --- | --- | --- | --- | --- | --- | --- | --- |
|  | **Intake** | | **Swallowing** | | **Energy/**  **appetite** | | **Total score** | |
| **Dysphagia Handicap Index (DHI)** | ***r*** | ^a)^ | ***r*** | ^a)^ | ***r*** | ^a)^ | ***r*** | ^a)^ |
| Physical | .041 | Weak | .336 | Moderate | .306 | Moderate | .345 | Moderate |
| Functional | .225 | Weak | .237 | Weak | .314 | Moderate | .389 | Moderate |
| Emotional | -.056 | Weak | .344 | Moderate | .158 | Weak | .225 | Weak |
| Total score | .121 | Weak | .338 | Moderate | .246 | Weak | .376 | Moderate |
| **DHI self-rated** | -.266 | Weak | .323 | Moderate | .147 | Weak | .037 | Weak |
| **Activities of daily living (Barthel index)** | -.826 | Strong | -.217 | Weak | -.066 | Weak | -.613 | Strong |
| **Dysphagia (4QT)** | .001 | Weak | .511 | Moderate | .503 | Moderate | .521 | Moderate |
| **Volume-viscosity swallow test (V-VST)** |  |  |  |  |  |  |  |  |
| Dysphagia | .401 | Moderate | .237 | Weak | -.220 | Weak | .275 | Weak |
| Viscosity | .348 | Moderate | -.189 | Weak | .036 | Weak | .158 | Weak |
| Volume | .137 | Weak | .425 | Moderate | -.077 | Weak | .254 | Weak |
| **Penetration-Aspiration Scale (PAS)** | -.127 | Weak | -.082 | Weak | -.006 | Weak | -.166 | Weak |
| **Functional Oral Intake Scale (FOIS)** | -.095 | Weak | -.184 | Weak | .089 | Weak | -.164 | Weak |

^a)^ Spearman correlations: weak = *r* <0.3; moderate = *r* 0.3-0.6; strong = *r* >0.6

Table 2. Correlations between Minimal Eating Observation Form – version II (MEOF-II) and different clinical characteristics among patients with Chronic obstructive pulmonary disease (n=25).

|  | **MEOF-II** | | | | | | | |
| --- | --- | --- | --- | --- | --- | --- | --- | --- |
|  | **Intake** | | **Swallowing** | | **Energy/**  **appetite** | | **Total score** | |
| **Dysphagia Handicap Index (DHI)** | ***r*** | ^a)^ | ***r*** | ^a)^ | ***r*** | ^a)^ | ***r*** | ^a)^ |
| Physical | .334 | Moderate | .506 | Moderate | .424 | Moderate | .553 | Moderate |
| Functional | .432 | Moderate | .501 | Moderate | .627 | Strong | .632 | Strong |
| Emotional | .022 | Weak | .533 | Moderate | .329 | Weak | .424 | Moderate |
| Total score | .331 | Moderate | .477 | Moderate | .445 | Moderate | .522 | Moderate |
| **DHI self-rated** | .184 | Weak | .456 | Moderate | .296 | Weak | .446 | Moderate |
| **Activities of daily living (Barthel index)** | -.657 | Strong | -.412 | Moderate | -.386 | Moderate | -.567 | Moderate |
| **Dysphagia (4QT)** | .236 | Weak | .664 | Moderate | .533 | Moderate | .678 | Strong |
| **Volume-viscosity swallow test (V-VST)** |  |  |  |  |  |  |  |  |
| Dysphagia | .298 | Weak | .132 | Weak | .333 | Moderate | .326 | Moderate |
| Viscosity | .450 | Moderate | -.053 | Weak | .337 | Moderate | .185 | Weak |
| Volume | .563 | Moderate | .320 | Moderate | .406 | Moderate | .523 | Moderate |
| **Penetration-Aspiration Scale (PAS)** | .253 | Weak | .150 | Weak | .236 | Weak | .266 | Weak |
| **Functional Oral Intake Scale (FOIS)** | -.152 | Weak | -.136 | Weak | -.253 | Weak | -.249 | Weak |

^a)^ Spearman correlations: weak = *r* <0.3; moderate = *r* 0.3-0.6; strong = *r* >0.6

Table 3. Correlations between Minimal Eating Observation Form – version II (MEOF-II) and different clinical characteristics among patients with Multiple Sclerosis (n=24).

|  | **MEOF-II** | | | | | | | |
| --- | --- | --- | --- | --- | --- | --- | --- | --- |
|  | **Intake** | | **Swallowing** | | **Energy/**  **appetite** | | **Total score** | |
| **Dysphagia Handicap Index (DHI)** | ***r*** | ^a)^ | ***r*** | ^a)^ | ***r*** | ^a)^ | ***r*** | ^a)^ |
| Physical | .534 | Moderate | .563 | Moderate | .446 | Moderate | .579 | Moderate |
| Functional | .440 | Moderate | .435 | Moderate | .588 | Moderate | .580 | Moderate |
| Emotional | .419 | Moderate | .294 | Weak | .419 | Moderate | .466 | Moderate |
| Total score | .614 | Moderate | .575 | Moderate | .618 | Strong | .692 | Strong |
| **DHI self-rated** | .516 | Moderate | .450 | Moderate | .638 | Strong | .634 | Strong |
| **Activities of daily living (Barthel index)** | -.557 | Moderate | -.342 | Moderate | -.349 | Moderate | -.491 | Moderate |
| **Dysphagia (4QT)** | .601 | Strong | .557 | Moderate | .482 | Moderate | .672 | Strong |
| **Volume-viscosity swallow test (V-VST)** |  |  |  |  |  |  |  |  |
| Dysphagia | .000 | Weak | .480 | Moderate | .375 | Moderate | .380 | Moderate |
| Viscosity | -.132 | Weak | .174 | Moderate | -.215 | Weak | .000 | Weak |
| Volume | .110 | Weak | .435 | Moderate | .347 | Moderate | .376 | Moderate |
| **Penetration-Aspiration Scale (PAS)** | .413 | Moderate | .342 | Moderate | .369 | Moderate | .349 | Moderate |
| **Functional Oral Intake Scale (FOIS)** | -.392 | Moderate | -.322 | Moderate | -.355 | Moderate | -.328 | Moderate |

^a)^ Spearman correlations: weak = *r* <0.3; moderate = *r* 0.3-0.6; strong = *r* >0.6

Table 4. Correlations between Minimal Eating Observation Form – version II (MEOF-II) and different clinical characteristics among patients with Parkinsons disease (n=26).

|  | **MEOF-II** | | | | | | | |
| --- | --- | --- | --- | --- | --- | --- | --- | --- |
|  | **Intake** | | **Swallowing** | | **Energy/**  **appetite** | | **Total score** | |
| **Dysphagia Handicap Index (DHI)** | ***r*** | ^a)^ | ***r*** | ^a)^ | ***r*** | ^a)^ | ***r*** | ^a)^ |
| Physical | .163 | Weak | .444 | Moderate | .185 | Weak | .439 | Moderate |
| Functional | .301 | Moderate | .576 | Moderate | .341 | Moderate | .587 | Moderate |
| Emotional | .273 | Weak | .628 | Strong | .058 | Weak | .466 | Moderate |
| Total score | .312 | Moderate | .620 | Strong | .202 | Weak | .571 | Moderate |
| **DHI self-rated** | .430 | Moderate | .422 | Moderate | .284 | Weak | .549 | Moderate |
| **Activities of daily living (Barthel index)** | -.713 | Strong | -.475 | Moderate | .017 | Weak | -.573 | Moderate |
| **Dysphagia (4QT)** | .303 | Moderate | .537 | Moderate | .048 | Weak | .436 | Moderate |
| **Volume-viscosity swallow test (V-VST)** |  |  |  |  |  |  |  |  |
| Dysphagia | -.008 | Weak | .008 | Weak | .178 | Weak | .185 | Weak |
| Viscosity | -.144 | Weak | .198 | Weak | .084 | Weak | .062 | Weak |
| Volume | .420 | Moderate | .147 | Weak | -.074 | Weak | .309 | Moderate |
| **Penetration-Aspiration Scale (PAS)** | .010 | Weak | .375 | Moderate | .120 | Weak | .186 | Weak |
| **Functional Oral Intake Scale (FOIS)** | -.022 | Weak | -.374 | Moderate | -.127 | Weak | -.189 | Weak |

^a)^ Spearman correlations: weak = *r* <0.3; moderate = *r* 0.3-0.6; strong = *r* >0.6
